# Economic Burden and Clinical Epidemiology of Dialysis: A Cross-Sectional Analysis of Chronic Kidney Disease Patients

**DOI:** 10.7759/cureus.93372

**Published:** 2025-09-27

**Authors:** Moiz N Butt, Usman Zafar, Farrukh Ansar, Sundas Butt, Abdul Basit, Muhammad Abrar, Syed A Abrar, Ahmed Faraz, Sammar Ahmed, Sultan Awan

**Affiliations:** 1 Internal Medicine, Noble's Hospital, Douglas, GBR; 2 Department of Medicine, Alkhidmat Raazi Hospital, Rawalpindi, PAK; 3 Internal Medicine, Addenbrooke's Hospital, Cambridge University Hospitals NHS Foundation Trust, Cambridge, GBR; 4 Accident and Emergency, Diana Princess of Wales Hospital, Grimsby, GBR; 5 Internal Medicine, Noble’s Hospital, Douglas, GBR

**Keywords:** chronic kidney disease (ckd), economic burden of healthcare, hemodialysis, patients’ out-of-pocket costs, renal failure

## Abstract

Introduction: Chronic kidney disease (CKD) represents a rising global health burden, with millions requiring dialysis as a form of kidney replacement therapy. In low- and middle-income countries such as Pakistan, dialysis care is partially subsidised, but patients often face substantial out-of-pocket (OOP) costs, creating financial strain.

Methods: This descriptive, cross-sectional study was conducted at a dialysis unit of a secondary care hospital in Pakistan. All patients on maintenance hemodialysis were invited to participate; the response rate was 80.8%. Data were collected through structured questionnaires and review of medical records. Demographic, clinical, and economic variables were captured, including comorbidities, dialysis duration and frequency, use of supportive therapies, and direct, indirect medical and non-medical costs in 2024 US dollars (USD). Statistical analysis was done using IBM SPSS Statistics version 26.0 (IBM Corp., Armonk, NY, USA).

Results: The cohort comprised 42 patients, 66.7% of whom were male (n = 28), with a mean CKD duration of 6.8 ± 5.2 years and a median dialysis duration of 1.0 year (IQR, 0.6-2.0 years). The mean age of the cohort was 58.3 ± 13.7 years (range, 25-83 years). Most (95.2%) were on twice-weekly hemodialysis. Despite full coverage of dialysis sessions through the Sehat Sahulat Programme, all patients incurred recurrent OOP costs. The median monthly “basic bundle” (consultation, labs, medications) cost was Pakistani rupees (PKR) 22,100 (approximately USD $78.40), equivalent to a mean annual cost of PKR 316,057 ± 149,030 (USD $1,121.55 ± $528.92). Additional costs included erythropoietin (85.7%, median PKR 7,600/month), blood transfusions (42.9%, median PKR 12,000/year), hospitalisations (38.1%, median PKR 190,000/year), transport (median PKR 4,800/month), and special diet (57.1%, median PKR 10,000/month). Using cumulative burden thresholds, 28.6% of households exceeded 100% of their monthly household income for expanded costs (4.8% for basic costs).

Conclusion: Dialysis patients in Pakistan carry a heavy dual burden of disease and economic hardship. While sessional dialysis is publicly financed, significant ancillary costs remain, often exceeding household income and driving catastrophic health spending. Policies expanding financial protection to include medications, laboratory testing, nutrition, and transport are urgently needed.

## Introduction

Chronic kidney disease (CKD) is a mounting global health crisis, affecting over 673 million individuals worldwide as of 2021. This corresponds to 8.5% to 9.8% of the global population [1]. The prevalence increased by 29.3% since 1990, although the age-standardised prevalence remained stable (+1.2%) [2]. CKD rose in the global rankings of causes of death from 17th in 1990 to 12th in 2017 and is expected to continue climbing in the coming years [2]. Literature suggests that CKD contributed to 1.4 million cardiovascular (CVD) deaths and 25.3 million CVD disability-adjusted life years (DALYs) in 2017 [2]. Overall, CKD and impaired kidney function together led to 2.6 million deaths globally (2.4-2.8 million) [2]. The burden is particularly striking in low‑ and middle‑income countries (LMICs), where over 80% of CKD-related morbidity occurs and access to kidney replacement therapy remains limited [3]. CKD is now the seventh leading risk factor for global mortality [3]. It is estimated that by 2040, CKD will be the fifth leading cause of years of life lost (YLLs) globally [3].

CKD imposes a considerable global economic burden, which increases dramatically once patients progress to kidney failure. At this stage, the requirement for kidney replacement therapy, which is either dialysis or renal transplantation, demands intensive healthcare resources, driving up treatment expenditures and creating a significant societal impact [4]. In addition, individuals with advanced CKD face an elevated risk of cardiovascular complications such as myocardial infarction, stroke, and heart failure, which further increase healthcare utilisation and compound the overall financial burden [5]. Dialysis, the most common form of kidney replacement therapy, imposes a substantial economic burden. Globally, annual costs per patient vary widely, with averages of approximately USD 49,900 to 57,300 for hemodialysis and USD 43,000 to 49,500 for peritoneal dialysis in high-income countries reported in 2023 [6].

In resource-constrained settings, cost pressures are even more acute. A review published in 2024 analysing eight studies conducted in Asian countries estimated mean annual dialysis costs around USD 23,358, while USD 4,977 for general CKD management [7]. In Pakistan specifically, an estimate from 2023 reported that hemodialysis has been estimated to cost between USD 1,680 and USD 2,000 per annum, which often exceeds the household income of affected families, most of whom rely on a single breadwinner earning between USD 110 and USD 500 per month [8]. Despite CKD’s rising prevalence and cost burden, Pakistan’s health system remains under-resourced [9]. The country spends approximately 1.2% percent of GDP on health, and more than half of healthcare expenditure continues to be out-of-pocket [10]. Although state-run initiatives, such as the Sehat Sahulat Programme, provide some healthcare coverage [11]. But many CKD patients continue to face significant and recurring out-of-pocket costs for consultations, laboratory tests, medications, and supportive therapies like erythropoietin, dietary supplements, and transport, exacerbating financial hardship, medical debt, and asset depletion.

This study aims to bridge a key gap in the literature by presenting a clinical epidemiologic profile of dialysis patients in Pakistan, combined with a detailed economic burden assessment. We examine patient demographics, comorbidity prevalence, CKD and dialysis duration, and treatment patterns, alongside direct medical and non‑medical costs, income loss, household income, and coping strategies. By integrating epidemiological description with an economic lens, this cross‑sectional analysis delivers critical insights for policymakers, clinicians, and health economists. Findings will inform resource allocation, guide financial protection strategies, and support interventions aiming to reduce both the clinical and economic hardship of CKD patients in low-resource settings.

## Materials and methods

Study design and setting

This was a descriptive, cross-sectional study conducted at Alkhidmat Raazi Hospital, a tertiary care centre in Rawalpindi, Punjab, Pakistan. The study was conducted from March to August 2025. The study was designed to evaluate the clinical epidemiologic profile and economic burden of patients receiving maintenance hemodialysis.

Study population and sampling

All patients receiving maintenance hemodialysis at the outpatient dialysis unit were invited to participate in the study. Of the 52 eligible patients, 42 (80.8%) provided informed consent and were therefore included in the final analysis. Participants were enrolled consecutively. Inclusion criteria were: age ≥18 years, established diagnosis of CKD with at least two months on maintenance dialysis, and willingness to provide informed consent. Patients who were critically ill at the time of recruitment, had previously undergone kidney transplantation, or declined participation were excluded.

Data collection

Data were collected through a combination of a structured, interviewer-administered questionnaire and a review of patients’ medical records. The questionnaire was predesigned to capture a broad range of variables covering demographic, clinical, and economic domains, while the medical records were used to validate clinical details and dialysis-related history. Demographic information included age, sex, marital status, educational attainment, employment status, and household size, providing insight into the socioeconomic context of each participant. Clinical variables encompassed the duration since CKD diagnosis, length of time on dialysis, frequency of dialysis sessions, and the presence of comorbidities such as hypertension, diabetes mellitus, ischemic heart disease, and anaemia. Additionally, use of supportive therapies was documented, including erythropoietin (EPO) administration, blood transfusions, and hospital admissions during the preceding year. Economic data were collected with particular attention to the multidimensional costs of care. Direct medical costs included expenditures on nephrologist consultations, routine laboratory investigations, medications, and dialysis-related supplies. Direct non-medical costs were also recorded, such as transport to and from dialysis centers and the additional expenses incurred for special dietary requirements. Finally, indirect costs were assessed by documenting self-reported loss of income due to absenteeism from work, the need to liquidate household assets, and the presence of outstanding medical debt.

Data analysis

All data were entered into Microsoft Excel (Redmond, WA, USA) and analysed using IBM SPSS Statistics version 26.0 (IBM Corp., Armonk, NY, USA). Continuous variables were summarised as means with standard deviations (SD) or medians with interquartile ranges (IQR), depending on data distribution. As cost data were highly skewed, we confirmed non-normal distribution through the Shapiro-Wilk tests and visual inspection of histograms. Therefore, costs were primarily reported as medians with interquartile ranges, supplemented by means with SD for comparability. Categorical variables were presented as frequencies and percentages. Comparisons between earners and non-earners were performed using the Mann-Whitney U test for non-parametric continuous variables. Chi-square and Fisher’s exact tests were done for categorical variables. A p-value <0.05 was considered statistically significant. Cost data were recorded in Pakistani rupees (PKR) and converted to US dollars (USD) using the prevailing exchange rate (1 USD = PKR 281.80 at the time of analysis). All costs were standardised to 2024 price levels and reported in both PKR and 2024 USD to ensure comparability with other studies.

Ethical considerations

Ethical approval for this study was obtained from the Institutional Review Board (IRB) of Alkhidmat Raazi Hospital, Rawalpindi, Pakistan (Approval No: IRB/A/05/24). All participants provided written informed consent before data collection. Confidentiality and anonymity were ensured throughout the study in accordance with the Declaration of Helsinki.

## Results

Sociodemographic and clinical characteristics

A total of 42 patients were included, of whom 66.7% (n = 28) were male. The mean age of the cohort was 58.3 ± 13.7 (range 25-83). The majority were married (61.9%, n = 26), and the most common primary language was Punjabi (52.4%, n = 22). Nearly all (95.2%, n = 40) had at least one comorbidity; 52.4% (n = 22) reported three and 9.5% (n = 4) reported four coexisting comorbidities. The most prevalent individual comorbidities were anaemia (95.2%, n = 40), hypertension (85.7%, n = 36), diabetes mellitus (47.6%, n = 20), and ischemic heart disease (23.8%, n = 10). The median time since CKD diagnosis was five years (IQR 3.0-10.0; mean 6.8 ± 5.2, range 11 months-20 years). The median duration of dialysis was one year (IQR 0.6-2.0; mean 1.5 ± 1.2, range 2 months-5 years). Most patients (95.2%, n = 40) were on a twice-weekly dialysis schedule and 4.8% (n = 2) thrice-weekly, with a median of eight sessions/month (IQR 8-8; mean 8.2).

Patients were categorised as income earners (n = 14, 33.3%) and non-earners (n = 28, 66.7%) for subgroup comparisons. There were no statistically significant differences between groups in baseline demographic or clinical characteristics (all p > 0.05; Table 1).

**Table 1:** Baseline demographic and clinical characteristics of dialysis patients by earning status (N = 42) Data are shown as N (%) for categorical and mean ± SD or median [IQR] for continuous variables. χ² tests for categorical and Mann–Whitney U tests for continuous variables. p < 0.05 is significant. CKD: chronic kidney disease

Variable	Category	Overall N (%)	Earners n=14	Non-earners n=28	Test	p
Gender	Male	28 (66.7)	10 (71.4)	18 (64.3)	χ²(1)=0.20	0.65
	Female	14 (33.3)	4 (28.6)	10 (35.7)		
Marital status	Married	26 (61.9)	10 (71.4)	16 (57.1)	χ²(2)=1.26	0.53
	Widowed	10 (23.8)	2 (14.3)	8 (28.6)		
	Single	6 (14.3)	2 (14.3)	4 (14.3)		
Primary language	Punjabi	22 (52.4)	9 (64.3)	13 (46.4)	χ²(3)=2.23	0.53
	Urdu	16 (38.1)	4 (28.6)	12 (42.9)		
	Pashto	2 (4.8)	1 (7.1)	1 (3.6)		
	Hindko	2 (4.8)	0 (0.0)	2 (7.1)		
Education	No formal	6 (14.3)	1 (7.1)	5 (17.9)	χ²(5)=3.64	0.60
	Primary	10 (23.8)	3 (21.4)	7 (25.0)		
	Secondary	10 (23.8)	2 (14.3)	8 (28.6)		
	Higher secondary	6 (14.3)	2 (14.3)	4 (14.3)		
	Graduation	8 (19.0)	4 (28.6)	4 (14.3)		
	Post-grad	2 (4.8)	2 (14.3)	0 (0.0)		
Dialysis duration	<1 year	18 (42.9)	4 (28.6)	14 (50.0)	χ²(2)=2.47	0.29
	1–3 years	18 (42.9)	7 (50.0)	11 (39.3)		
	3–6 years	6 (14.3)	3 (21.4)	3 (10.7)		
CKD duration (years)		6.8 ± 5.2	7.1 ± 5.5	6.7 ± 5.1	U = 190	0.79

Direct medical and non-medical costs

All patients were covered by the state-funded Sehat Sahulat Programme, but still incurred out-of-pocket costs (Table 2). The universal monthly bundle (nephrologist fee, laboratory tests, medications) cost a median PKR 22,100 [PKR 18,500-29,500] (USD $78.42), with a mean 26,338 ± 12,419 (USD $93.46 ± $44.09), equivalent to a mean annual cost of PKR 316,057 ± 149,030 (USD $1,121.55 ± $528.92).

**Table 2:** Direct medical and non-medical costs of dialysis patients by earning status (N = 42) Data are presented as mean ± SD, median [IQR], and N (%). Group comparisons between earners and non-earners were performed using Mann–Whitney U tests. p < 0.05 is considered statistically significant.

Item	Utilization	Unit	Overall Mean ± SD	Median [IQR]	Earners Median [IQR]	Non-earners Median [IQR]	U	p
Nephrologist fee	42 (100%)	Monthly	850 ± 0	850 [850–850]	850 [850–850]	850 [850–850]	—	—
Labs	42 (100%)	Monthly	7,650 ± 0	7,650 [7,650–7,650]	7,650 [7,650–7,650]	7,650 [7,650–7,650]	—	—
Medications	42 (100%)	Monthly	17,838 ± 12,419	13,600 [10,000–21,000]	17,500 [12,000–22,500]	13,000 [9,500–19,000]	142	0.21
Basic total	42 (100%)	Monthly	26,338 ± 12,419	22,100 [18,500–29,500]	26,300 [20,000–33,000]	21,000 [17,000–28,000]	138	0.18
Basic total	42 (100%)	Annual	316,057 ± 149,030	265,200 [222,000–354,000]	315,600 [240,000–396,000]	252,000 [204,000–336,000]	138	0.18
Erythropoietin (EPO)	36 (85.7%)	Monthly	8,394 ± 5,578	7,600 [4,000–10,000]	9,000 [6,000–12,000]	7,000 [3,500–9,000]	110	0.09
Blood transfusions	18 (42.9%)	Annual	15,444 ± 16,550	12,000 [7,000–15,000]	14,000 [8,000–15,000]	12,000 [6,500–15,000]	126	0.33
Hospital admissions	16 (38.1%)	Annual	335,000 ± 319,541	190,000 [97,500–487,500]	210,000 [120,000–520,000]	170,000 [80,000–480,000]	120	0.25
Transport	42 (100%)	Monthly	7,010 ± 4,736	4,800 [4,000–8,000]	6,500 [4,500–9,500]	4,000 [3,000–7,000]	100	0.04*
Extra diet	24 (57.1%)	Monthly	13,333 ± 6,945	10,000 [9,500–16,250]	15,000 [12,000–18,000]	9,500 [7,500–12,000]	95	0.02*

Among additional medical costs, 85.7% (n = 36) required EPO with a median monthly cost of PKR 7,600 [PKR 4,000-10,000] and a mean annual cost of PKR 100,733 ± 66,933. Blood transfusions were reported by 42.9% (n = 18; median PKR 12,000/year) and hospital admissions by 38.1% (n = 16; median PKR 190,000/year). Direct non-medical costs included transport (median PKR 4,800 [PKR 4,000-8,000]/month) and extra diet (57.1%, median PKR 10,000 [PKR 9,500-16,250]/month).

Compared with non-earners, earners had significantly higher transport (U = 100, p = 0.04) and extra diet (U = 95, p = 0.02) expenditures; other cost categories were not significantly different (Table 2).

Household income and cost burden

Only 14 (33.3%) patients were income earners. Earners had substantially higher per-capita income (median PKR 30,833 [PKR 22,071-44,508]) than non-earners (median PKR 10,000 [PKR 6,607-14,250]) and higher household income (median 150,000 vs 70,000; U = 302, p = 0.0047). Household sizes were similar (median 6; U = 202, p = 0.88). We analysed monthly dialysis-related costs as a share of household income across cumulative thresholds, separately for basic and expanded cost definitions. Among basic costs, earners were significantly more likely to remain below the lowest burden threshold (<10%) compared with non-earners (28.6% vs 0.0%; χ² = 8.08, p = 0.004), while proportions converged at higher thresholds (Table 3).

**Table 3:** Basic costs of households below each burden threshold by earning status *p < 0.05 statistically significant. Data are shown as N (%) of households below each threshold (earners n = 14; non-earners n = 28). Chi-square (χ²) tests compared proportions between groups at each threshold.

Threshold (share of income)	Earners n=14 (%)	Non-earners n=28 (%)	χ²(1)	p
<10%	4 (28.6)	0 (0.0)	8.08	0.004*
<25%	8 (57.1)	12 (42.9)	0.88	0.35
<50%	12 (85.7)	20 (71.4)	1.12	0.29
<75%	12 (85.7)	26 (92.9)	0.75	0.39
<90%	12 (85.7)	26 (92.9)	0.75	0.39
<100%	14 (100.0)	26 (92.9)	1.05	0.31
≥100%	0 (0.0)	2 (7.1)	1.03	0.31

For expanded costs, earners were consistently more likely to fall below lower thresholds (<10% to <50%), whereas non-earners more frequently exceeded the highest threshold (≥100%) (35.7% vs 14.3%; χ² = 4.15, p = 0.04) (Table 4). Among those whose own income did not fully cover dialysis-related costs (n = 32, 76.2%), all reported receiving support from family or friends; 14 (33.3%) had sold assets, and six (14.3%) reported outstanding medical debt.

**Table 4:** Expanded costs of households below each burden threshold by earning status *p < 0.05 statistically significant. Data are shown as n/N (%) of households below each threshold (earners n = 14; non-earners n = 28). Chi-square (χ²) tests compared proportions between groups at each threshold.

Threshold (share of income)	Earners n=14 (%)	Non-earners n=28 (%)	χ²(1)	p
<10%	2 (14.3)	0 (0.0)	4.24	0.04*
<25%	6 (42.9)	0 (0.0)	14.14	0.0002*
<50%	10 (71.4)	8 (28.6)	6.96	0.008*
<75%	10 (71.4)	16 (57.1)	0.99	0.32
<90%	12 (85.7)	16 (57.1)	3.86	0.049*
<100%	12 (85.7)	18 (64.3)	2.17	0.14
≥100%	2 (14.3)	10 (35.7)	4.15	0.04*

## Discussion

In this cross-sectional analysis of 42 maintenance hemodialysis patients, we found a striking comorbidity burden. It was found that anaemia (95.2%) and hypertension (85.7%) were the most prevalent comorbidities coupled with predominantly twice-weekly dialysis and substantial, recurring out-of-pocket expenditures despite state coverage of dialysis sessions. The “basic bundle” which included a nephrologist visit, laboratory tests, and medications alone, accounted for a median PKR 22,100/month. Besides that, many patients have to bear EPO, transfusions, hospitalisations, transport, and diet-related costs. Most households could not fully cover dialysis-related expenses from income, and their coping strategies included reliance on family support, asset liquidation, and medical debt. These findings synthesise the clinical profile and treatment intensity with the economic pressure points that patients face in a low-resource setting.

The clinical profile aligns with regional observations that end-stage kidney disease (ESKD) patients often present late, accumulate multiple cardiometabolic comorbidities, and require resource-intensive regimens [12]. Contemporary guidance underscores that anaemia is nearly ubiquitous in advanced CKD and should be approached systematically, identifying correctable causes, individualising erythropoiesis-stimulating agent (ESA) initiation, and avoiding high haemoglobin targets that elevate cardiovascular risk [13]. The Kidney Disease: Improving Global Outcomes (KDIGO) 2025 anaemia guideline emphasises evaluation for correctable causes before ESA or hypoxia-inducible factor prolyl hydroxylase inhibitors (HIF-PHI) therapy and ongoing reassessment after major health events [14]. Our high EPO utilisation rate (85.7%) and nontrivial transfusion frequency (42.9%) highlight both clinical need and the additive costs of supportive therapy.

Dialysis frequency in our cohort was largely twice weekly (95.2%). While thrice-weekly hemodialysis remains the default in high-resource settings [15]. The incremental hemodialysis (including twice-weekly schedules) has been proposed for patients with significant residual kidney function, provided adequacy targets are met and close monitoring is feasible [16]. In resource-constrained environments, incremental regimens can be used; however, they must not substitute for adequacy, symptom control, and outcome monitoring [17]. Our data showed a median of eight sessions per month. This data should be interpreted in light of this trade-off between economics and adequacy, and future work should incorporate residual kidney function, clearance metrics, and hospitalisation outcomes by frequency.

The economic signals in our study resonate with the literature [18]. Even where dialysis is nominally free, ancillary expenses such as medications, laboratory testing, transport, and special diet can be substantial and recurrent [19]. A South Asian regional study including Pakistan estimated annual hemodialysis costs near US$4,873 with 51 to 75% out-of-pocket (OOP), reflecting how incomplete coverage shifts costs to households [20]. Qualitative and mixed-methods work from Pakistan similarly documents that the totality of direct and indirect costs often exceeds household income, forcing difficult choices and treatment interruptions [21]. Our findings that many households rely on family transfers, sell assets, or incur debt are consistent with these reports and indicate persistent financial toxicity. At the health-system level, Pakistan’s financing structure exacerbates vulnerability. OOP spending constitutes a large share of current health expenditure, indicating shallow prepayment and limited financial protection [22]. This macro context provides the backdrop for our cohort’s cost profile and helps explain why even a state-financed dialysis session may not translate into full financial protection for patients.

Interpreting our “burden vs income” thresholds through standard financial-protection lenses strengthens external validity. The WHO budget-share and capacity-to-pay approaches define catastrophic health spending at thresholds such as >10% of total household consumption/income and >40% of non-food (capacity-to-pay) expenditure [23]. Applying similar cumulative thresholds, our table shows a substantive fraction of households crossing 25% and 50% marks for monthly costs, and a notable minority exceeding 100% of monthly household income. This is an extreme expression of financial catastrophe in the dialysis context.

The Sehat Sahulat Programme has expanded access to high-cost services and draws favourable satisfaction ratings among both patients and clinicians [24]. However, patient-level studies report low card utilisation in some communities and persistent OOP for items outside the benefits package [25]. Our data are concordant: dialysis sessions were covered, but medications (notably ESAs), labs, transport, and diet remained household liabilities. Program adjustments that bundle essential ancillaries (iron therapy, ESAs under clinical criteria from KDIGO, routine labs, and travel stipends) could meaningfully reduce residual OOP and protect continuity of care. Hospital admissions represented a major cost tail in our cohort (median PKR 190,000/year among those admitted). Preventive gains may be achievable by investing in nutrition therapy, infection prevention, and vascular access quality [26]. Kidney Disease Outcomes Quality Initiative (KDOQI) highlights individualised medical nutrition therapy as central to outcomes and resource use, while vascular-access guidelines emphasise strategies that reduce catheter dependence and access complications, both pathways that can curb hospitalisation frequency and costs [27]. Embedding dietitian support and access surveillance into benefit packages may thus yield clinical and economic dividends [27].

Taken together, our data support several policy and practice implications. First, move beyond session-only financing: covering essential ancillaries (iron, ESAs with KDIGO-aligned criteria, labs, access procedures) and offsetting transport are likely to reduce catastrophic spending and improve adherence. Second, incremental hemodialysis should be formalized with adequacy monitoring and clear criteria for escalation to thrice-weekly schedules. Finally, integrating financial-protection metrics such as share of households exceeding 10% to 25% thresholds into routine program monitoring would align dialysis policy with universal health coverage (UHC) goals and enable timely benefit-package tuning [28].

Strengths and limitations

Our study has multiple strengths and limitations. Strengths include the integration of clinical epidemiology with a granular cost inventory spanning direct medical, direct non-medical, and indirect costs, and the use of standardized threshold analysis to interpret burden versus income. Limitations include the single-centre design and modest sample size, which restrict statistical power and limit broader generalizability. These findings should therefore be interpreted as exploratory, highlighting important trends and justifying the need for larger, multi-centre studies to confirm and extend these observations. The study cohort was drawn from a single secondary-care dialysis unit and, therefore, may not be fully representative of all dialysis patients in Pakistan. Nonetheless, the demographic and socioeconomic profile observed here aligns with previously published national data, supporting partial external validity. We did not collect detailed demographic or clinical information for the 10 patients who declined participation, and therefore cannot formally compare responders and non-responders. This could introduce selection bias, although the participation rate (80.8%) was high. Price levels and exchange rates are time-sensitive; while we standardized to a single period, inflation dynamics may affect generalizability. In line with reporting standards, all costs were inflated to 2024 USD to enable comparability across studies. Future research should include multi-site sampling, prospective cost diaries, and linkage to outcomes such as hospitalizations, access complications, mortality to quantify how specific benefit-package components influence clinical and financial trajectories.

## Conclusions

This study highlights the dual burden of chronic kidney disease in Pakistan, marked by a high prevalence of comorbidities, alongside significant out-of-pocket expenditures despite partial state coverage of dialysis sessions. Patients faced substantial residual costs for medications, investigations, transport, and dietary needs, often exceeding household income and leading to asset sales and debt. These findings underscore the urgent need for policy interventions that move beyond dialysis session financing to include essential supportive therapies, nutritional care, and transport assistance. Strengthening financial protection within programs like the Sehat Sahulat Programme, while exploring cost-effective strategies such as peritoneal dialysis expansion and monitored incremental hemodialysis, could mitigate both clinical risks and economic hardship. Integrating these measures into routine kidney care is critical to advancing equitable, sustainable, and patient-centered dialysis services in low-resource settings.
